# Permittivity measurements for raw and boiled quinoa seeds versus temperature, bulk density, and moisture content

**DOI:** 10.1016/j.crfs.2023.100528

**Published:** 2023-06-02

**Authors:** Rafael Pérez-Campos, José Fayos-Fernández, Juan Monzó-Cabrera

**Affiliations:** a Departamento de Tecnologías de la Información y las Comunicaciones, Universidad Politécnica de Cartagena, 30202, Cartagena, Spain

**Keywords:** Quinoa, Permittivity, Resonant technique, Dielectric constant, Loss factor

## Abstract

Quinoa is a nutrient-rich pseudocereal that is gaining popularity in European countries since it is gluten-free and an interesting source of fat, proteins, minerals, and amino acids. However, up to date, the electric permittivity of quinoa seeds has not been measured and, therefore, this hampers the possibility of designing optimized recipes for its microwave processing. In this work, the permittivity of both raw and boiled quinoa seeds is measured around 2.45 GHz at several conditions for temperature, moisture content, and bulk density. The grain kernel permittivity is also estimated from the Complex Refractive Index (CRI) mixture equation and different bulk density measurements. The obtained results show different temperature behaviours for raw and boiled seeds, whereas the permittivity of quinoa seeds versus moisture content and bulk density was as expected: the permittivity (both the dielectric constant and loss factor) levels increased as the observed variables did. From the measured data, it can be concluded that both raw and boiled quinoa can be processed with microwave technology, although care must be taken with raw quinoa grain kernels since the permittivity increases significantly with temperature, and a thermal runaway may occur.

## Highlights

• Quinoa permittivity is affected by temperature, bulk density, and moisture content.
• Estimation of grain kernel permittivity from bulk density permittivity dependency.
• Determining dielectric properties of granular materials using cavity-resonator technology.
• Different permittivity behaviours versus temperature for raw and boiled quinoa.

## Graphical abstract

**Figure:**
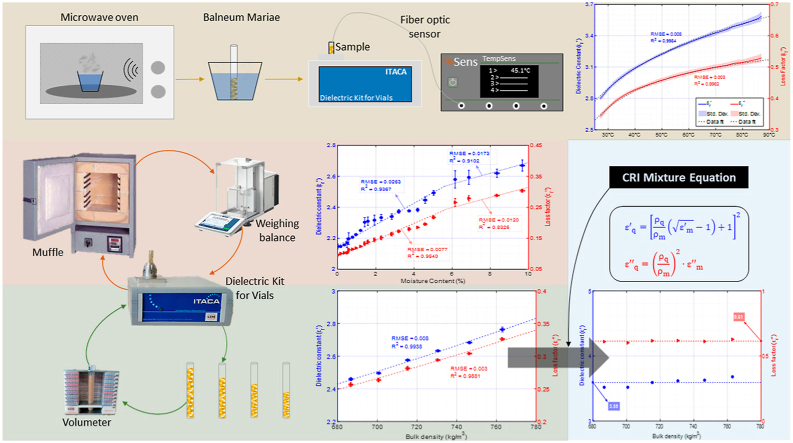

## 1 Introduction

Quinoa (Chenopodium quinoa Willd) belongs to the group of pseudocereals that have similar nutritional characteristics to cereals (Rosa et al., 2020). This pseudograin has very interesting properties such as drought and frost resistance (Carmen, 1984), mineral storage such as magnesium, zinc, iron, manganese, and potassium (Rosa et al., 2020), the production of high yields even under adverse conditions (Borges et al., 2003), low or no gluten content, and a higher amount of proteins, lysine, and fat in comparison with common cereals (Vilche et al., 2003; Jan et al., 2019).

In addition, the cultivation of quinoa is easily adaptable to different weather conditions and world regions (Herencia et al., 1999; Jacobsen et al., 1994). Quinoa crops can be an alternative to conventional cereal plantations, and consequently, the physical properties of quinoa grains should be characterized for further storage, transportation, and transformation. In fact, quinoa is now a common product at food stores in Spain, and it is likely to be distributed as a new conventional or microwaveable prepared meal.

Some physical properties of quinoa seeds have been measured in previous studies. For instance, Vilche et al. (2003) have determined the sphericity, the density, the porosity, the angle of repose, the static coefficient of friction, and the terminal velocity for quinoa seeds in the moisture range of 4.6–25.8% (dry basis). Rosa et al. have measured the length, width, thickness, specific weight, and water content of both amaranth and quinoa seeds (Rosa et al., 2020). The effect of moisture content on true density, coefficient of friction, sphericity, bulk density, length, diameter, porosity, angle of repose, initial cracking force, rupture force, and rupture energy is studied in (Jan et al., 2019) for two different germplasms of Indian quinoa. The moisture diffusivity of quinoa has been determined during convective drying versus air temperature and initial moisture content (Gely and Santalla, 2007).

It is well known that the moisture content influences the volume of kernels and their surface area (Vilche et al., 2003), which are important during processes such as aeration and the modelling of grain drying. Furthermore, higher moisture content values of grains can cause a noticeable rise in pressure on silo walls, and variations in moisture content can also cause flow issues in silos, e.g., erratic flows (Kibar et al., 2010). Thus, the determination of the moisture content of quinoa grain kernels is crucial for adequately designing machines and storage facilities.

Regarding the interaction of quinoa with microwaves, the effects of some heat treatments on the structural and functional properties of quinoa have been investigated (Wang et al., 2021), concluding that microwave heating and boiling are recommended for isolating the quinoa protein as a basis for food products. Microwave heating was also applied to quinoa proteins in (Huang et al., 2022), and the obtained results indicated that microwaves facilitated the production of protein aggregates and improved the gel strength while maintaining the water-holding capacity. Several processing methods for obtaining puffed quinoa have been compared (Zapana et al., 2020), concluding that gun and extrusion puffing achieves better outcomes than microwave heating. Microwave drying of quinoa and amaranth seeds was applied at several irradiation densities (Hernández-Maqueda et al., 2018) to assess the drying time, germination rate, and field survival of the seeds. The results indicated that the germination rates of quinoa seeds were more affected than those of amaranth seeds. The authors also indicated that, in the case of quinoa seeds, the temperature had to be monitored and controlled carefully.

The effect of the electromagnetic fields on the materials is determined by their dielectric properties. These properties can be expressed in terms of the complex relative permittivity ($\varepsilon_{r}$), hereafter referred to as permittivity, which is the absolute permittivity normalized to the permittivity of free space ($\varepsilon_{0}=8.854\cdot 10^{-12}F/m$). The permittivity is a complex quantity represented as $\varepsilon_{r}=\varepsilon_{r}^{\prime }-j\varepsilon_{r}^{\prime \prime }$, where $\varepsilon_{r}^{\prime }$ is the well-known dielectric constant and $\varepsilon_{r}^{\prime \prime }$ the so-called loss factor. The dielectric constant, which is not constant at all because of its dependence on moisture content, temperature, frequency, and density, is responsible for the amount of electric energy that can be stored within the irradiated material. The loss factor characterizes the ability of the material to dissipate microwave energy into heat.

Determining dielectric properties for all types of materials has been a long-studied topic in the field of microwave applications. When microwaves are used to treat materials for various purposes, such as pasteurization (Linand Wang, 2020), drying (Zeng et al., 2022), rice bran stabilization (Ling et al., 2018), and others, a thorough understanding of the material's dielectric properties becomes critical. Microwaves can be used to extract saponins from quinoa with high efficiency (Gianna et al., 2012). Other researchers recently discovered that microwave processing provides the highest antioxidant potency composite index (Sharma et al., 2022). However, the efficiency of the microwave processing is highly dependent on its relative permittivity and the microwave applicator design.

Despite the increasing interest in quinoa and the application of microwave processes to this pseudocereal, little data is known about its permittivity. The only electromagnetic data for quinoa has been found in (Rosa et al., 2020), where the authors provide static dielectric constant data for both amaranth and quinoa by using the capacitance meter of a multimeter and a cylindrical capacitive sensor. Thus, characterization of the dielectric constant and loss factor as a function of various parameters (such as temperature, bulk density, and moisture content) must be conducted to provide the required information for the further design of high-efficient microwave applicators in terms of energy. In addition, the data provided in this paper can also be utilized when designing sensors for non-destructive moisture content estimation for granular quinoa at frequencies around 2.45 GHz, and it can also be useful in simulating dielectric heating for granular quinoa microwave processing as well as providing a theoretical basis for quinoa dehydration under dielectric heating.

Nevertheless, to the best of the authors’ knowledge, there are not any works in the literature that provide high-frequency data near the 2.45 GHz industrial, scientific, and medical (ISM) frequency band. In this work, the permittivity of raw and boiled quinoa seeds has been measured around 2.45 GHz for different conditions of water content, temperature, and bulk density. The estimation of the permittivity of the quinoa grain kernel is also provided by using well-known expressions and data from the literature and bulk density-dependent measurements.

## 2 Materials and methods

### 2.1 Materials

Raw Peruvian quinoa (Chenopodium quinoa Willd) seeds were used, which were manufactured by Laboratorios Almond S.L. and were commercially available at a well-known Spanish supermarket chain. Both raw quinoa and boiled quinoa were tested. The preparation procedure for the boiled quinoa began with the raw quinoa seeds immersed in boiling water for 10 min. According to (Sharma et al., 2022), the water absorption capacity of 10-min boiled quinoa seeds is nearly as high as that of longer-boiled quinoa seeds, and therefore 10 min was selected as the boiling time. Afterwards, the seeds were extracted from the recipient and left to dry for 24 h in the shade. Due to water absorption, the quinoa seed mass was multiplied by 3.2 as a result of the boiling process.

**Fig. 1:**
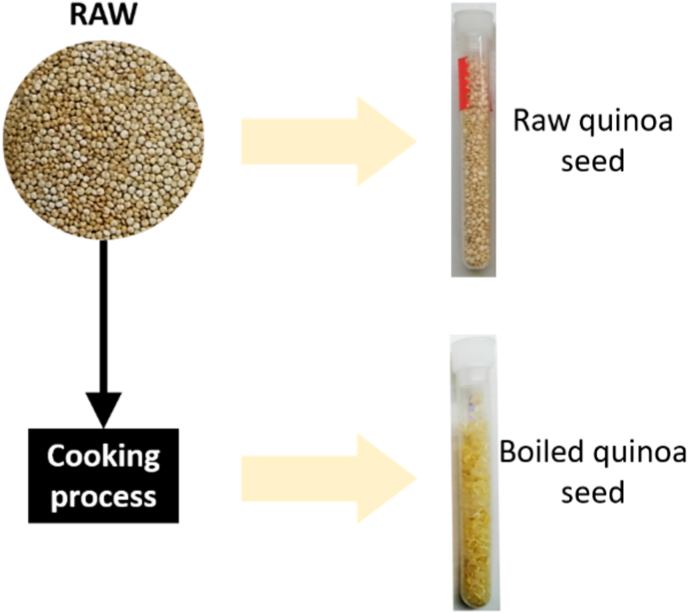
Example of raw and boiled quinoa samples.

For the preparation of the quinoa samples, 6-mL quasi-cylindrical polypropylene test tubes (Deltalab Ref. 400400) were used as sample containers. The internal diameter and height of the test tubes were 10.3 mm and 86.9 mm, respectively. Fig. 1 shows the schema of the raw and boiled quinoa seeds sample preparation.

The quinoa mass of the samples used for the different permittivity measurements is shown in Table 1. The initial conditions of the samples for the different dependencies on temperature, bulk density, and moisture content studied in this work are also shown in that table. The averaged values per sample set are shown in Table 1.

**Table 1:** Quinoa samples conditions for each permittivity measurement test.

Dependence analysis test	No. Of samples	Mass (g)	Temperature (°C)	Bulk density (kg/m^3^)	Moisture Content (%)
Temperature	3x Raw	3.72 ± 0.01	[27, 87]	740 ± 10	9.7 ± 0.4
	3x Boiled	2.80 ± 0.07	[24, 85]	580 ± 10	263 ± 9
Bulk density	3x Raw	3.43 ± 0.01	24 ± 2	[690, 760]	9.7 ± 0.4
	3x Boiled	2.77 ± 0.02	23 ± 2	[510, 830]	263 ± 9
Moisture content	3x Raw	3.72 ± 0.13^a^	24 ± 2	750 ± 10^a^	[0, 9.7]
	3x Boiled	2.83 ± 0.06^a^	23 ± 2	580 ± 10^a^	[0, 263]

a Values at initial conditions. These values were reduced after every drying process cycle due to water content evaporation.

It should be noted that boiled quinoa grains can be easily compacted to higher levels compared to raw quinoa due to their lower hardness and higher malleability.

### 2.2 Size analysis of the quinoa seeds

Ten seeds were drawn at random from the bulk sample to determine the size and shape of the quinoa seeds. A digital micrometer (Preisser Digi-Met, with a least count of 0.001 mm) was used to measure three primary dimensions for each individual seed: length (L), width (W), and thickness (T). The expression described by equation (1) was used to calculate the equivalent diameter ($D_{eq}$) as the geometric mean of the three dimensions.

(1) $$D_{eq}=\sqrt[{3}]{L\cdot W\cdot T}$$

Sphericity (Ø) is defined as the ratio of the surface area of a sphere with the same volume as the seed to the surface area of the seed. Thus, the higher the sphericity value, the closer the grain's shape is to a sphere. The sphericity was calculated from the equivalent diameter using the equation below (Jan et al., 2019).

(2) $$Ø=\frac{D_{eq}}{L}$$

### 2.3 Permittivity measurement equipment

The relative electric permittivity measurements were performed with a Dielectric Kit for Vials (DKV) manufactured by the ITACA research institute of the Universidad Politécnica de Valencia (Spain). This instrument provides a microwave frequency-swept stimulus into a resonant cavity and measures the reflected power as a response. It requires a prior calibration of the empty cavity before measuring the response of the cavity loaded with the sample. The resonance shifting (displacement of the curve's peak location) and its widening due to the decrease of the cavity quality factor when a dielectric material is inserted into its resonant structure (Sheen, 2009) are computed to determine the dielectric constant and loss factor of liquid, granular, or powdered materials around 2.45 GHz. The relationship between complex permittivity and resonance characteristics is obtained by using numerical methods based on mode-matching and circuit analysis, as reported in (Gutiérrez-Cano et al., 2020).

The operating frequency of the DKV ranges from 1.5 to 2.6 GHz. It can characterize materials with dielectric constants lower than 100 and loss factors ranging from 0.001 to 15. This equipment provides 1% and 5% uncertainty for the determination of the dielectric constant and the loss factor, respectively. The repeatability and linearity values provided by the manufacturer are around 0.2%.

The measured resonant frequencies for the raw quinoa seeds were 2.51 ± 0.01 GHz and 2.31 ± 0.02 GHz for the boiled quinoa seeds. Thus, the relative electric permittivity data provided at several temperatures, moisture level contents, and bulk density conditions is near the 2.45 GHz ISM band.

### 2.4 Test protocol for the analysis of the temperature dependence of the quinoa permittivity

The permittivity measurement of quinoa seeds as a function of temperature was performed in a similar way to that used in (Pérez-Campos et al., 2020). To minimize the air gaps within the samples, the test tubes were filled slowly while being wobbled.

**Fig. 2:**
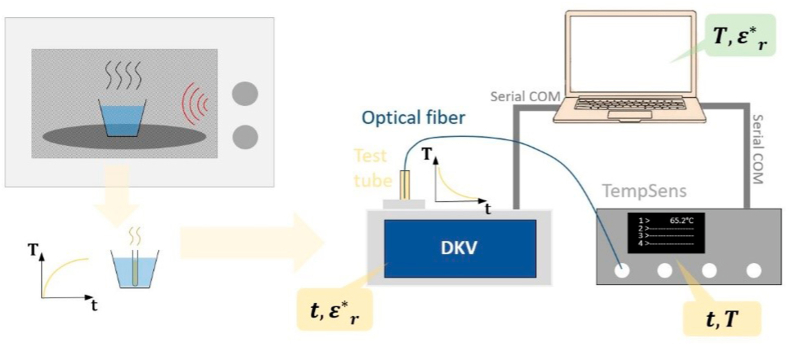
Scheme of the procedure and setup for measuring the complex permittivity dependence on temperature.

The temperature evolution of the sample was monitored using an optical fibre as a sensor and the TempSens signal conditioner equipment from OpSens, as depicted in Fig. 2. The TempSens provides an accuracy of ±0.8 °C and ±0.3 °C for temperatures higher and lower than 45 °C, respectively.

Initially, each sample was heated to nearly 90 °C in a water bath (Balneum Mariae), using a 150 mL glass of water pre-heated in a domestic microwave oven. Afterwards, the heated sample was inserted into the DKV equipment to monitor its permittivity while cooling down. Thus, its permittivity is measured while cooling. The temperature over time T(t) logging was carried out by running an in-house LabView code on a computer connected to the TempSens equipment. Simultaneously, the instrument software provided by the DKV manufacturer was used to log the sample permittivity over time $\varepsilon_{r}\left(t\right)$. The same computer was used to log both measurements, and therefore they had the same time reference to combine the data and provide the temperature dependence of the permittivity $\varepsilon_{r}\left(T\right)$ using a Matlab script, as reported in (Fayos-Fernández et al., 2015).

Every sample was measured once. The modelled discrete-temperature dependency of the permittivity data of the same kinds of samples (raw or boiled quinoa) was computed as the average of their 3-set samples, with a discretized resolution of ΔT=1°C. That means that all the measured permittivity values within the same temperature bin T±ΔT/2 were averaged. In this way, the first discrete temperature was set to 87 °C, matching the bin boundaries of [86.5, 87.5]°C, and the last one was set to 27 °C, matching the bin boundaries of [26.5, 27.5]°C.

Regarding the moisture content and the bulk density, they were kept constant during the experiments, as shown in Table 1.

### 2.5 Protocol for testing the bulk density dependence of quinoa permittivity

Another study carried out in this work consisted of measuring permittivity dependence versus bulk density.

The mass of the quinoa samples was kept constant as stated in Table 1, while the volume was reduced from 5.0 to 4.5 cm^3^ for the raw quinoa and from 5.4 to 3.4 cm^3^ for the boiled grains. The mass sample was measured by means of a weighing scale from Mettler Toledo, model XPR56DR, with an accuracy of ±0.01 mg. The sample volume was obtained by using an in-house volumeter designed with a parallax error control, as shown in (Fayos-Fernández et al., 2018).

The minimum bulk density obtained for raw quinoa seeds was 690 ± 20 kg/m^3^, whereas the maximum bulk density was 760 ± 20 kg/m^3^. Regarding the boiled seeds, those values were 510 ± 10 kg/m^3^ for the minimum bulk density and 810 ± 30 kg/m^3^ for the maximum. The permittivity versus bulk density results were computed as the average of three samples per bulk density value.

For the raw quinoa samples employed in these density-dependent measurements, the average dry-basis moisture content was around 10%. This value was 285% for the boiled seeds, as indicated in Table 1. During all bulk density-dependent measurements, the sample temperature matched the room temperature.

### 2.6 Measurement procedure for moisture content-dependent complex permittivity of quinoa seeds

The dielectric properties versus the moisture content of raw and boiled quinoa seeds were also measured in this work. Three samples of both raw and boiled quinoa seeds were prepared. Then, the samples were gradually dried in a muffle furnace at 90 °C for periods of 2 h. After each drying period, the samples were withdrawn until they cooled down to room temperature. The amount of evaporated water was known by weighting the samples on a Mettler Toledo precision scale (model XPR56DR), while their dielectric properties were measured in the DKV. This procedure was repeated until all the grains within the test tubes were assumed to be completely dried. Given the good resolution of the weighing scale (0.01 mg), small decrements in mass were always detected between two consecutive measurements. Therefore, it was considered that the end of the process was achieved when mass changes were less than 3 mg during three consecutive weigh-ins (around 0.1% of the sample initial mass). The fact that there was no detectable permittivity variation between the two most recent measurements validates this threshold. The latest mass sample obtained has been considered the dry mass reference.

The moisture content (X) employed in this work has been expressed as a dry basis as shown in equation (3):

(3) $$X=\frac{m_{i}-m_{d}}{m_{d}}$$

where $m_{i}$ and $m_{d}$ are the sample mass for a given moisture content and the sample mass when the sample is completely dried, respectively. Consequently, X is the ratio of the water mass contained in the quinoa seeds versus the dried solid mass since $m_{i}-m_{d}$ represents the water mass in the sample. The results of the moisture content-dependent complex permittivity values were calculated by averaging the measurements from three different samples.

### 2.7 Calculations for raw quinoa grain kernel permittivity estimation

Granular bulk materials, such as quinoa, are a mixture of grain kernels and the air that fills the space between them. Thus, when measuring the permittivity of this kind of material as a bulk, one must keep in mind that the measured effective permittivity is the result of a coupled combination of the permittivities of grain kernel and air. Therefore, the measurement results depend on the density of the granular material. Consequently, when studying the permittivity of quinoa, it is of utmost importance to establish a reliable relationship between the bulk density of the air-kernel mixture and the measured permittivity.

The relationship between the permittivity and the bulk density of certain granular materials has been previously reported. For instance, Kent worked with fish meal at 9.78 GHz (Kent, 1977); Klein analysed that relationship for granular coal (Klein, 1981); and Nelson studied it on several granular and powdered materials such as pulverized coal, limestone, plastics, and granular wheat and flour (Nelson, 1983, 1984, 2005). Trabelsi et al. created a calibration function independent from bulk density that allows for the estimation of the moisture content in granular materials from permittivity measurements (Trabelsi et al., 1998; Nelson, 2008). In other studies, authors determined the bulk density of the material prior to studying its dielectric properties (Torrealba-Meléndez et al., 2015).

In this contribution, the relationship between bulk density and the permittivity of both raw and boiled quinoa seeds is established. Additionally, the permittivity of the quinoa grain kernel is estimated by applying the dielectric mixture equations for a two-phase mixture, thus calculating the permittivity of a solid material (quinoa seed) from the permittivity of a granular mixture (seed-air).

In the literature, several dielectric mixture equations have been used to evaluate the dielectric properties of an air-kernel mixture. Some examples are the Bruggeman-Hanai, Maxwell-Garnett, CRI, or Landau and Lifshitz-Looyenga (LLL) equations. Nonetheless, Nelson investigated their reliability and discovered that the CRI and LLL equations provided more accurate predictions than the other mixture equations (Nelson, 2005). We compared both CRI and LLL equations and found that lower RMSE values were offered by the CRI model for the loss factor, whereas similar RMSE values were obtained for the dielectric constant. As a result, the CRI mixture equation was selected to estimate the permittivity of the quinoa grain kernel.

For this purpose, it is necessary to know the permittivity of the air-grain mixture and its bulk density ($\rho_{m}$), as well as the quinoa grain kernel density ($\rho_{q}$). The latter was previously estimated in (Vilche et al., 2003), and it ranged from 928 to 1188 kg m^−3^. The Complex Refractive Index (CRI) mixture equation for a two-phase mixture (Nelson, 2005) is used in this work to estimate the quinoa kernel permittivity, $\varepsilon_{q}$:

(4) $$\sqrt{\varepsilon_{m}}=v_{a}\sqrt{\varepsilon_{a}}+v_{q}\sqrt{\varepsilon_{q}}$$

where $\varepsilon_{m}$ is the permittivity of the air-quinoa mixture (bulk grain), $\varepsilon_{a}$ is the air permittivity ($\varepsilon_{a}=1$), $\varepsilon_{q}$ is the permittivity of the quinoa kernel, and $v_{a}$ and $v_{q}$ are the volume fractions of air and quinoa ($v_{a}+v_{q}=1$), respectively. Both $v_{a}$ and $v_{q}$ can be obtained from the bulk density of the mixture and the quinoa kernel density ($v_{a}=\frac{\rho_{m}}{\rho_{q}}$). Hence, equation (4) can be expressed as shown in equations (5), (6):

(5) $$\varepsilon_{q}^{\prime }={\left[\frac{\rho_{q}}{\rho_{m}}\left(\sqrt{\varepsilon_{m}^{\prime }}-1\right)+1\right]}^{2}$$

(6) $$\varepsilon_{q}^{\prime \prime }={\left(\frac{\rho_{q}}{\rho_{m}}\right)}^{2}\cdot \varepsilon_{m}^{\prime \prime }$$

where $\varepsilon_{q}^{\prime }$ and $\varepsilon_{q}^{\prime \prime }$ are the dielectric constant and the loss factor of the quinoa grain kernel, respectively, and $\varepsilon_{m}^{\prime }$ and $\varepsilon_{m}^{\prime \prime }$ are the dielectric constant and loss factor of the mixture quinoa seeds and air, respectively.

### 2.8 Statistical analysis

All permittivity measurements were repeated three times. The standard deviation of the average value of triplicates was used to calculate error bars for all data points. Matlab was used to process the data (version R2019a, MathWorks software) as well as plot the data.

## 3 Results

The size of raw quinoa seeds as well as the measurements of the dielectric properties of both raw and boiled quinoa seeds are shown and discussed in this section versus different conditions of temperature, bulk density, and moisture content. The dielectric constant is represented with a blue colour and the loss factor is represented with a red colour. The data fits are represented by a dashed line.

### 3.1 Raw quinoa seeds

#### 3.1.1 Size and sphericity

A micrometer was used to measure the size of quinoa seeds, which have the appearance of a smoothed sphere. Because of the round longitudinal section, the seed's major dimensions, length and width, are approximately equal, as shown in Table 2. The equivalent diameter and sphericity were calculated as described in equations (1), (2), respectively.

**Table 2:** Size and sphericity of quinoa seeds at 9.7% dry-basis moisture content.

L (mm)	W (mm)	T (mm)	$D_{eq}$ (mm)	Ø
2.18 ± 0.32	2.13 ± 0.24	1.05 ± 0.16	1.69 ± 0.20	0.78 ± 0.04

#### 3.1.2 Complex permittivity dependence on temperature

**Fig. 3:**
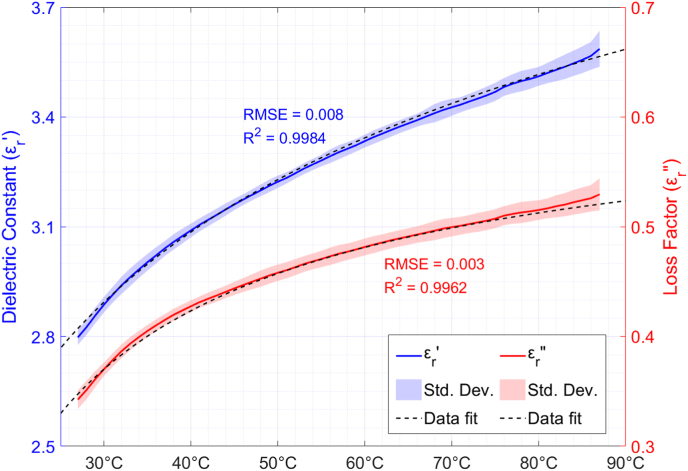
Temperature dependence of permittivity of raw quinoa and exponential data fitting. $\rho_{m}$ = 740 kg/m^3^; X = 9.7%.

The average behaviours versus temperature of the dielectric constant and loss factor of raw quinoa samples are shown in Fig. 3 for a temperature range between 27 and 87 °C. The standard deviations of both magnitudes and their exponential fitting are also depicted in Fig. 3. From the obtained results, one can observe that both the dielectric constant and the loss factor are increasing with increasing sample temperatures. For the whole temperature range, the increments of the dielectric constant and the loss factor were around 28.6% and 60%, respectively.

An exponential function was used to fit the experimental data on raw quinoa's complex permittivity dependence on temperature. The dielectric constant and the loss factor dependence on temperature data fittings are described by equations (7), (8), respectively:

(7) $$\varepsilon_{r}^{\prime }\left(T\right)=7.59-7.69\cdot T^{-0.145}$$

(8) $$\varepsilon_{r}^{\prime \prime }\left(T\right)=0.64-3.64\cdot T^{-0.765}$$

where T is the sample temperature in °C. The root mean square error (RMSE) and the coefficient of determination (R^2^) for the dielectric constant are 0.008 and 0.9984, respectively. For the loss factor, the RMSE is 0.003 and the R^2^ is 0.9962.

#### 3.1.3 Complex permittivity dependence on bulk density

**Fig. 4:**
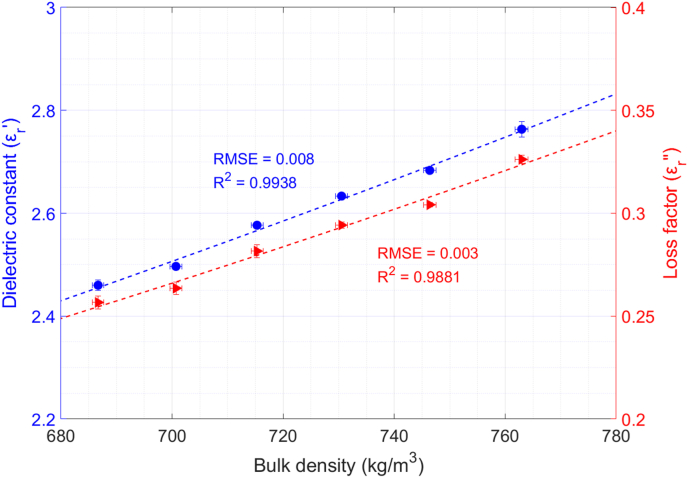
Permittivity dependence on bulk density of raw quinoa and polynomial data fitting. T = 24 °C; X = 9.7%.

The average values of the dielectric constant and the loss factor of raw quinoa samples versus bulk density are shown in Fig. 4. Standard deviation values are also shown with error bars. As can be observed, higher permittivity values are found as bulk density increases. Regarding the permittivity variation, the dielectric constant increment is around 12.2% for the studied range, and the loss factor rise is 26.9%. Given the granular form of the raw quinoa seeds and their hardness, it was not possible to make the measurements for a wider range of bulk densities.

According to Kent (1977), the relationship between permittivity and bulk density should be a quadratic function. Thereby, we have obtained equations (9), (10) for quinoa seeds by fitting second-order polynomials to dielectric constant and loss factor data:

(9) $$\varepsilon_{r}^{\prime }\left(\rho_{m}\right)=2.46\cdot {\rho_{m}}^{2}+0.43\cdot \rho_{m}+1$$

(10) $$\varepsilon_{r}^{\prime \prime }\left(\rho_{m}\right)=0.70\cdot {\rho_{m}}^{2}-0.11\cdot \rho_{m}$$

where $\rho_{m}$ is the bulk density of the sample (g/cm^3^). The RMSE and the R^2^ for the fitting of the dielectric constant are 0.008 and 0.9938, respectively, whereas for the fitting of the loss factor, the RMSE is 0.003 and the R^2^ is 0.9881.

#### 3.1.4 Moisture content-dependent complex permittivity

**Fig. 5:**
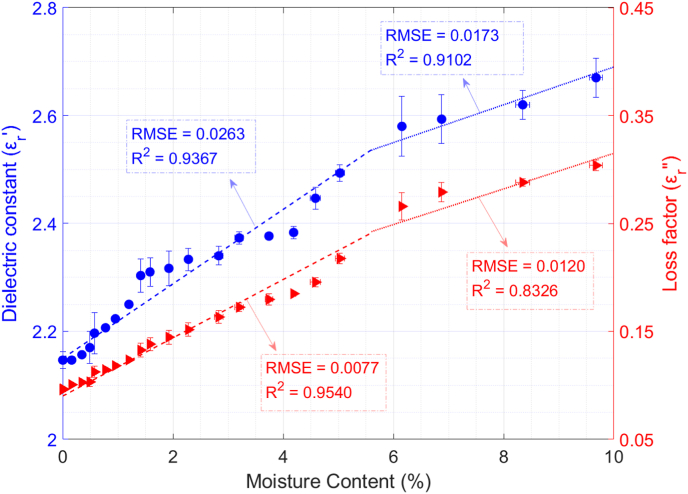
Permittivity behaviour of raw quinoa versus moisture content and polynomial data fitting. $\rho_{m}$ = 740 kg/m^3^; T = 24 °C.

Fig. 5 shows the variation of the dielectric constant and the loss factor versus dry-basis moisture content. Different parts can be distinguished in Fig. 5 both for dielectric constant and loss factor similar to those found in (Castro-Giráldez et al., 2012) for amaranth seeds. For moisture content values lower than 1%, the internal water seems to be strongly bound to quinoa tissue, and therefore, the mobility of water is reduced. This fact and the low amount of water are the primary reasons for the particularly low loss factor values. However, as the moisture content increases (i.e., the amount of water increases), so does its mobility, thereby causing an increment in the values of the loss factor and dielectric constant. Nevertheless, further research has to be conducted to test these assumptions.

The experimental data of permittivity evolution versus moisture content was interpolated by using a 2-piecewise function. The linear fittings for the real and imaginary parts of the permittivity are shown in equations (11), (12):

(11) $$\varepsilon_{r}^{\prime }\left(X\right)=\left\{ \begin{array}{cc} 6.90\cdot X+2.15 & ;X\in [0,5[\;\%\\ 3.50\cdot X+2.34 & ;X\in \left[5,10\right]\;\% \end{array}\right.$$

(12) $$\varepsilon_{r}^{\prime \prime }\left(X\right)=\left\{ \begin{array}{cc} 2.70\cdot X+0.09 & ;X\in [0,5[\;\%\\ 1.65\cdot X+0.15 & ;X\in \left[5,10\right]\;\% \end{array}\right.$$

where X is the dry-basis moisture content as calculated in equation (3). Regarding the first range (i.e., from 0 to 5%), the RMSE and R^2^ values for the fitting of the dielectric constant were 0.0263 and 0.9367, respectively, and for the fitting of the loss factor, the RMSE was 0.0077 and the R^2^ was 0.9540. For the second range, those values were 0.0173 (RMSE) and 0.9102 (R^2^) for the dielectric constant and 0.0120 (RMSE) and 0.8326 (R^2^) for the loss factor.

#### 3.1.5 Raw quinoa grain kernel permittivity estimation

**Fig. 6:**
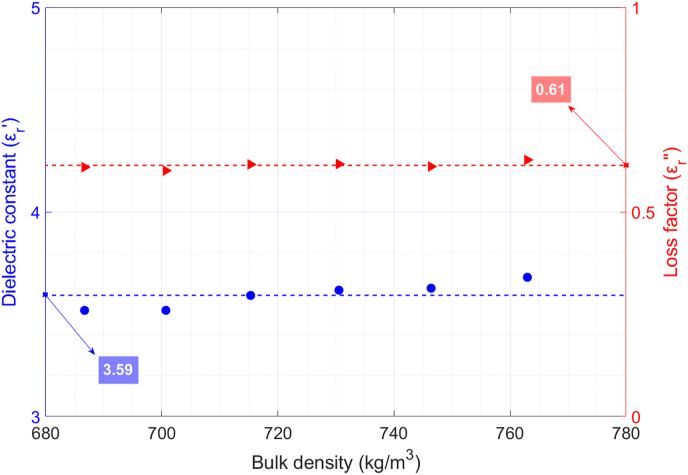
Estimation of average permittivity of quinoa grain kernel assuming $\rho_{k}=1058\;kg/m^{3}$ and using equations (5), (6). T = 24 °C; X = 9.7%.

Fig. 6 shows the permittivity of raw quinoa grain kernels, which has been estimated by using equations (5), (6), the average density of the quinoa grain kernel provided in (Vilche et al., 2003), $\rho_{k}=1058\;kg/m^{3}$, and the data of granular quinoa permittivity versus its bulk density previously depicted in Fig. 4. From the obtained results, one can observe that very similar permittivity values of quinoa grain kernels were estimated, although using different bulk densities. Small variations may be explained by small errors in the determination of the bulk density and the inherent error of permittivity measurements. An average value for the dielectric constant and the loss factor of the quinoa seeds has been obtained from the estimations in Fig. 6 and plotted as a constant.

In the case of the dielectric constant of the quinoa grain, $\varepsilon_{q}^{\prime }$, an average value of 3.59 and a standard deviation of 0.06, have been obtained. Regarding the loss factor of quinoa seeds, $\varepsilon_{q}^{\prime \prime }$, the calculated average value and its standard deviation were 0.61 and 0.01, respectively.

### 3.2 Boiled quinoa seeds

#### 3.2.1 Permittivity dependence on temperature

**Fig. 7:**
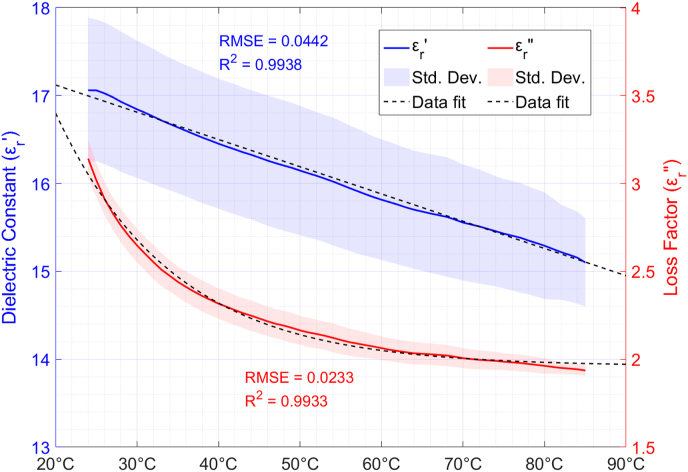
Permittivity variation of boiled quinoa seeds versus temperature and exponential data fitting. $\rho_{m}$ = 580 kg/m^3^; X = 263%.

Fig. 7 shows the permittivity variation versus temperature from 24 to 85 °C for the boiled quinoa samples. Both the dielectric constant and the loss factor values decrease with increasing sample temperatures, an opposite behaviour to that observed for raw quinoa seeds. A possible explanation for this difference is that the water absorbed by boiled seeds is mainly stored as free water, whereas the water in raw seeds is bound to the structure of the seeds, showing a different behaviour from free water. The dielectric constant and loss factor decrements are around 11.7% and 38.1%, respectively, for the whole temperature range.

A linear function fitting was employed for the experimental data on the temperature-dependent dielectric constant, whereas an exponential function fitting was employed for the loss factor, as described by equations (13), (14), respectively:

(13) $$\varepsilon_{r}^{\prime }\left(T\right)=-0.031\cdot T+17.74$$

(14) $$\varepsilon_{r}^{\prime \prime }\left(T\right)=1.96+5.77\cdot e^{-0.0694\cdot T}$$

where T is the sample temperature in °C. The RMSE and R^2^ values for the dielectric constant fitting are 0.0442 and 0.9938, respectively. For the loss factor fitting, the RMSE value is 0.023 and the R^2^ value is 0.9933.

#### 3.2.2 Permittivity dependence on bulk density

**Fig. 8:**
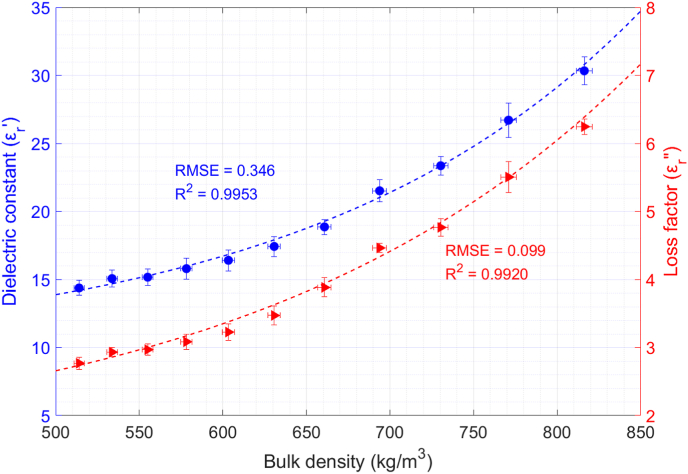
Permittivity variation of boiled quinoa versus bulk density of the sample and exponential data fitting. T = 23 °C; X = 263%.

Fig. 8 shows the permittivity variation of boiled quinoa seeds versus bulk density. One can notice that the larger the bulk density, the higher the permittivity values. The dielectric constant and loss factor increments are around 111% and 126%, respectively. In this case, a wider range of bulk density was studied since it was possible to compact the boiled seeds to a greater extent than in the case of raw seeds.

The experimental data for the bulk density-dependent complex permittivity of boiled quinoa seeds was modelled by an exponential function, as shown in equations (15), (16):

(15) $$\varepsilon_{r}^{\prime }\left(\rho_{m}\right)=9.56+0.35\cdot e^{5.03\cdot \rho_{m}}$$

(16) $$\varepsilon_{r}^{\prime \prime }\left(\rho_{m}\right)=1.37+0.15\cdot e^{4.30\cdot \rho_{m}}$$

where $\rho_{m}$ is the bulk density of the sample expressed in g/cm^3^. The RMSE is 0.346, and the R^2^ is 0.9953 for the dielectric constant fitting. Regarding the loss factor fitting, the RMSE is 0.099 and the R^2^ is 0.9920.

#### 3.2.3 Moisture content-dependent complex permittivity

**Fig. 9:**
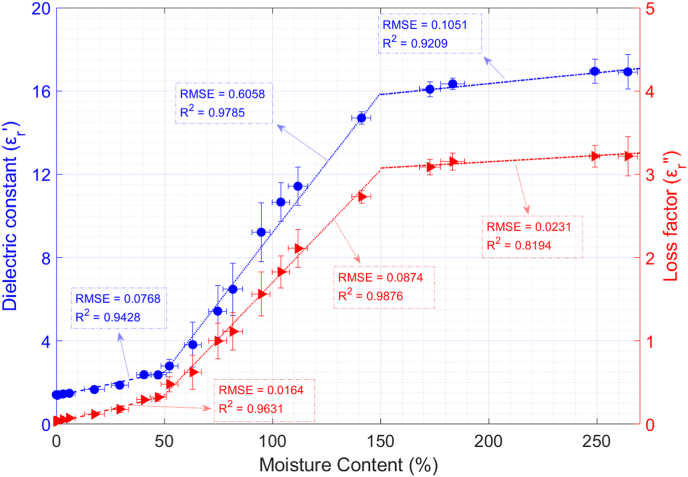
Permittivity dependence on moisture content for boiled quinoa and polynomial data fitting. ${\rho_{m}|}_{X=263\%}$ = 580 kg/m^3^; ${\rho_{m}|}_{X=0\%}$ = 340 kg/m^3^; T = 23 °C.

The dielectric constant and the loss factor variations versus dry-basis moisture content for boiled quinoa seeds are shown in Fig. 9. One can observe that both magnitudes highly decrease with decreasing moisture content values, mainly in the [50, 150] % moisture content range, whereas in the interval [150, 270] %, the permittivity decreases at a lower rate.

In this case, a 3-piecewise linear function was used to fit the experimental data on moisture content-dependent permittivity. The linear fitting for the dielectric constant is described by equation (17), and that for the loss factor is described by equation (18):

(17) $$\varepsilon_{r}^{\prime }\left(X\right)=\left\{ \begin{array}{c} \begin{array}{cc} 2.30\cdot X+1.35 & ;X\in [0,50[\;\% \end{array}\\ \begin{array}{cc} 13.41\cdot X-4.22 & ;X\in [50,150[\;\% \end{array}\\ \begin{array}{cc} 1.05\cdot X+14.25 & ;X\in \left[150,270\right]\;\% \end{array} \end{array}\right.$$

(18) $$\varepsilon_{r}^{\prime \prime }\left(X\right)=\left\{ \begin{array}{c} \begin{array}{cc} 0.63\cdot X+0.02 & ;X\in [0,50[\;\% \end{array}\\ \begin{array}{cc} 2.70\cdot X-1.00 & ;X\in [50,150[\;\% \end{array}\\ \begin{array}{cc} 0.15\cdot X+2.85 & ;X\in \left[150,270\right]\;\% \end{array} \end{array}\right.$$

where X is the dry-basis moisture content of the sample as calculated in equation (3). The RMSE values for the dielectric constant fitting are 0.0768 (0–50%), 0.6058 (50–150%), and 0.1051 (150–270%). On the other hand, the R^2^ values are 0.9428 (0–50%), 0.9785 (50–150%), and 0.9209 (150–270%). Regarding the loss factor, the RMSE values are 0.0164 (0–50%), 0.0874 (50–150%), and 0.0231 (150–270%), whereas the R^2^ values are 0.9631 (0–50%), 0.9876 (50–150%), and 0.8294 (150–270%).

## 4 Conclusions

In this work, the permittivity of both raw and boiled quinoa seeds has been measured for different conditions of temperature, bulk density, and moisture content. Important differences in permittivity values were observed for raw and boiled quinoa seeds due to the transformation of the internal structure of the quinoa and the significant increment of moisture content during the boiling process. More specifically, boiled quinoa seeds showed much higher values of permittivity than raw seeds due to the storage of free water during the cooking process.

The permittivity evolution versus temperature for raw and boiled seeds is very different as well. In the first case, the permittivity increased with increasing temperatures. However, in the second case, the permittivity decreased with increasing temperatures. As it has been explained above, a possible reason for this difference is the interactions of water with the surrounding structure: the little amount of available water in raw seeds is bound to its internal structure, but a great amount of free water is trapped within the grain kernel for boiled seeds. In fact, it is well known that free water permittivity decreases with increasing temperatures at frequencies lower than the water relaxation frequency (e.g., 2.45 GHz).

The permittivity behaviour versus bulk density and moisture content for both raw and boiling seeds showed higher dielectric constant and loss factor values with increasing bulk densities and moisture contents. However, the relationship between permittivity and these parameters was different, i.e., permittivity increases exponentially as a function of bulk density, whereas the permittivity dependence on moisture content shows various increasing slopes.

Despite the fact that several dielectric mixture equations have been utilized in the literature to examine the dielectric properties of an air-grain mixture, in this contribution, the average quinoa grain kernel permittivity has been determined using the CRI mixing equation and permittivity measurements at different bulk densities. The estimated raw grain kernel permittivity values were higher than the permittivity of the air-quinoa mixture, and low standard deviation values were obtained.

This research will contribute to scientific knowledge in the field of microwave quinoa treatment and will aid in understanding the effects of temperature, bulk density, and moisture content. The data presented in this paper can be used to develop sensors for the non-destructive determination of the moisture content of granular quinoa. Although the conclusions for quinoa permittivity should be considered valid only around the 2.45 GHz ISM frequency, they will reproduce at lower and higher microwave frequencies where dipolar rotation is the driving mechanism for dielectric losses.

## CRediT authorship contribution statement

**Rafael Pérez-Campos:** Term, Conceptualization, Software, Formal analysis, Investigation, Data curation, Writing – original draft, Visualization. **José Fayos-Fernández:** Methodology, Validation, Writing – review & editing. **Juan Monzó-Cabrera:** Conceptualization, Investigation, Resources, Writing – original draft, Writing – review & editing, Supervision.

## Declaration of competing interest

The authors declare that they have no known competing financial interests or personal relationships that could have appeared to influence the work reported in this paper.

## Data Availability

Data will be made available on request.
